# PCR-Based Molecular Diagnosis of Hepatitis Virus (HBV and HDV) in HCV Infected Patients and Their Biochemical Study

**DOI:** 10.1155/2016/3219793

**Published:** 2016-06-06

**Authors:** Muhammad Nasir Riaz, Muhammad Faheem, Muhammad Ayaz Anwar, Ummar Raheel, Yasmeen Badshah, Hashaam Akhtar, Kosar Tamanna, Muhammad Tahir, Najam us Sahar Sadaf Zaidi, Ishtiaq Qadri

**Affiliations:** ^1^Atta-ur-Rahman School of Applied Biosciences, National University of Sciences and Technology, Islamabad 44000, Pakistan; ^2^Department of Microbiology, Hazara University, Mansehra 21120, Pakistan; ^3^Ciencias Genomicas e Biotecnologia, Universidade Catolica de Brasilia, 70790-160 Brasilia, DF, Brazil; ^4^Laboratory of Biochemistry and Protein Chemistry, Cell Biology Department, University of Brasilia, 70910-900 Brasilia, DF, Brazil; ^5^King Fahd Medical Research Center, King Abdul Aziz University, Jeddah 21589, Saudi Arabia

## Abstract

Seroprevalence of HCV indicates that HCV is found in more than 10% of HBV- or HDV-infected patients worldwide leading to liver disease. Here we show HBV and HDV coinfection association with HCV infected Pakistani patients, study of disease severity, and possible interpretation of associated risk factors in coinfected patients. A total of 730 liver diseased patients were included, out of which 501 were found positive for HCV infection via PCR. 5.1% of patients were coinfected with HBV while 1% were coinfected with HBV and HDV both. LFTs were significantly altered in dually and triply infected patients as compared to single HCV infection. Mean bilirubin, AST, and ALT levels were highest (3.25 mg/dL, 174 IU/L, and 348 IU/L) in patients with triple infection while dual infection LFTs (1.6 mg/dL, 61 IU/L, and 74 IU/L) were not high as in single infection (1.9 mg/dL, 76 IU/L, and 91 IU/L). The most prominent risk factor in case of single (22%) and dual infection (27%) group was “reuse of syringes” while in triple infection it was “intravenous drug users” (60%). It is concluded that HBV and HDV coinfections are strongly associated with HCV infected Pakistani patients and in case of severe liver disease the possibility of double and triple coinfection should be kept in consideration.

## 1. Introduction

Chronic hepatitis is a common cause of liver related morbidity due to different hepatic viruses, where hepatitis B (HBV) and hepatitis C (HCV) have been identified as the main cause [1] and lead to many complications. Over a million persons die annually due to HBV related complications [2, 3]. HCV also leads to many complications including HCC in 32% of infected patients [4]. Infection with multiple viruses leads to management problems with higher incidence of morbidity and mortality [5]. Since hepatitis B, hepatitis C, and hepatitis D share almost the same modes of transmission, infection with more than one virus is possible [6]. Therefore, presence of dual and triple viral infections has been reported from various parts of the world [7]. 

HCV is a positive stranded RNA virus and has been classified into the genus* Hepacivirus* of the family Flaviviridae [8]. Lack of proper implementation of international standards in procedures like blood transfusion, reuse of injections, injecting drug users, tattooing, shaving from barbers, unsterilized dental reuse of needles for ear and nose piercing, and surgical instruments is the key factor of HCV transmission in Pakistan. Worldwide, there are about 170 million people infected with HCV, and three to four million individuals are diagnosed as new cases every year [9], while in Pakistan 10 million people are presumed to be HCV patients with 5% prevalence in general population [10].

Hepatitis B virus belongs to Hepadnaviridae family [11] with a circular genome of 3.2 kb composed of partially double-stranded DNA [12]. An average prevalence of hepatitis B antigen in Pakistan is 2.4% (range 1.4–11.0%) in healthy adults and 2.4% in pediatric population. The reuse of used syringes and unsafe blood transfusion are the major causes of spread of hepatitis B [10]. Hepatitis delta virus (HDV), first discovered by Rizzetto et al. [13] in a patient with chronic hepatitis B virus (HBV) infection, is a unique single-stranded negative circular RNA virus with genome of 1.7 kb that requires the helper function of HBV for infection [14]. It was originally thought to be a new nuclear antigen associated with HBV [5] but later proved to be a new virus that requires the surface antigens of HBV (HBsAgs) to support its life cycle and infectivity [15–17]. The prevalence of HDV accounts for 15–20 million people who are already infected with HBV [18]. Very limited data is available about the prevalence and epidemiology of HDV infection in Pakistan. HDV infection is present in 16.6% of hepatitis B infected patients in Pakistan [19]. In another study from Karachi 35.2% of the coinfection was reported. It was further explained that HBV/HDV coinfection resulted in the suppression of HBV DNA. A fair percentage of HBV/HDV coinfected patients with HBeAg negative had active hepatitis B infection and cirrhosis as compared to those with monoinfection [20]. Diagnosing multiple hepatitis viral infections is limited by low level of awareness among physicians and availability of simple diagnostic tests. Recently, good results were demonstrated by a single integrated protein microarray that could simultaneously determine in human sera two viral antigens (HBsAg and HBeAg) and seven antibodies (HBsAb, HBcAb, HBeAb, HCVAb, HDVAb, HEVAb, and HGVAb) of human hepatitis viruses within 20 minutes [21].

Treatment options are limited in triple infections as lamivudine alone or in combination with interferon has not shown much benefit in patients coinfected with HDV [22]. Since there is no report on triple infection caused by hepatitis viruses from Pakistani population [23], the aim of this study is to find the rate of coinfection of HBV and HDV in HCV infected Pakistani patients.

## 2. Material and Methods

### 2.1. Patients and Samples

Present study was conducted at Atta-ur-Rahman School of Applied Biosciences (ASAB), National University of Science and Technology (NUST), in collaboration with Capital Development Authority (CDA) Hospital, Islamabad, Railways Hospital (IIMCT), Rawalpindi, and Holy Family Hospital, Rawalpindi, Pakistan. The study was approved by the Institutional Review Board (IRB), ASAB, and NUST.

In the present study, a total of 730 patients, with liver disease, were included who were referred to ASAB diagnostics center during the period from July 2009 to March 2010. During the course of the study, venous blood samples were collected in the morning after 12-hour overnight fasting. 137 samples were collected from Capital Development Authority (CDA) Hospital, Islamabad, 257 samples from Government Holy Family Hospital, Rawalpindi, 155 samples from Railways Hospital (IIMCT), Rawalpindi, and 181 from Atta-ur-Rahman School of Applied Biosciences (ASAB) diagnostics.

#### 2.1.1. Extraction of HCV RNA

Sera of the patients were subjected to viral RNA extraction by using QIAamp viral RNA extraction kit (Qiagen), according to the manufacturer's protocol.

#### 2.1.2. Qualitative Analysis (PCR-Based Detection)

All ELISA positive samples with chronic hepatitis C infection were further confirmed for HCV presence by PCR. Indirect ELISA method is used for the detection of antibodies to HCV in two-step incubation procedure (microLISA).

#### 2.1.3. Detection of HCV RNA by Nested PCR

Viral RNA was taken to reverse-transcribe the 5′ NCR of HCV using Moloney murine leukemia virus reverse transcriptase (M-MLV RTase, Fermentas) in a total reaction volume of 20 *μ*L. The reaction mixture contained 4 *μ*L MMLV (5x) buffer, 1 *μ*L M-MLV reverse transcriptase (RT) enzyme, 1 *μ*L dNTPs (10 mM), 0.5 *μ*L Rnase inhibiter (Fermentas), 1.5 *μ*L RNase free water, 1 *μ*L specific antisense primer P2 5′-ACTCGCAAGCACCTATCAGGCAGTAC-3′ (Macrogen, Korea), and 10 *μ*L template (viral RNA). cDNA was synthesized using ABI Veriti 96-well thermocycler. Cycle conditions for cDNA were as follows: 42°C for 55 minutes followed by 70°C for 10 minutes. The cDNA produced was stored at 4°C for short-term storage or −20°C for prolonged storage. cDNA product was used for qualitative analysis of HCV infection. The first round PCR was performed using sense P1 5′-CCCTGTGAGGAACACTGTCTTCACGC-3′ and antisense P2 5′-ACTCGCAAGCACCTATCAGGCAGTAC-3′ primers (Macrogen, Korea) followed by second round PCR (nested PCR), using the first round product with inner sense P3 5′-GAAAGCGTCTAGCATGGCG-3′ and antisense P4 5′-CACAAGGCCTTTCCGACC-3′ primers (Macrogen, Korea). PCRs were carried out using Taq polymerase (Fermentas) for 35 cycles. The PCR product was visualized by 1.2% agarose gel and stored at −20°C until further use.

#### 2.1.4. Detection of HBsAg in Serum Samples from Patients with HCV Infection

The sera of HCV samples were tested for infection of HBV. To analyze the HBV infection, strip device (ACON, USA) was used. For detection of HBsAg sandwich ELISA method was used (microLISA).

### 2.2. HBV DNA Detection through PCR

For the detection of hepatitis B virus, the specific portion of surface gene was amplified through PCR from HBV genomic DNA using specific forward and reverse primers.

### 2.3. Viral DNA Extraction

Viral DNA was isolated from the serum of HBV infected patient's samples using Qiagen Kit (Germany) according to the manufacturer's protocol.

### 2.4. Polymerase Chain Reaction

The extracted DNA was subjected to PCR for the detection of HBV. 20 *μ*L PCR reaction mixture contain DNA, PCR Master Mix (50 units/mL of Taq DNA polymerase supplied in a proprietary reaction buffer pH 8.5, 400 *μ*M dATP, 400 *μ*M dGTP, 400 *μ*M dCTP, 400 *μ*M dTTP, and 3 mM MgCl_2_) (Promega Cat.# M7502), 10 mM of sense primer (Macrogen, Korea), 5′-CCGAATTCGCCACCATGCATCCTGCTGCTATGCCTCATCT-3′ and 10 mM of antisense primer (Macrogen, Korea), and 5′-CCCGAATTCGCCACCATGCGAACCACTGAACAAATGGCACT-3′. Thirty cycles of DNA amplification were performed. The condition of each cycle was denaturation at 94°C for 45 seconds, primer annealing at 64°C for 45 seconds, and elongation at 72°C for 45 seconds, followed by a final elongation at 72°C for 7 minutes. PCR product was further amplified with the inner nested primer set, 10 mM sense primer (Macrogen, Korea), 5′-CCCGAATTCGCCACCATGGGTATGTTGCCCGTTTGTCCTCT-3′, and 10 mM of antisense primer (Macrogen, Korea) 5′-CCCGAATTCGCCACCATGGGCACTAGTAAACTGAGCCA-3′ for an additional 30 cycles under the same reaction conditions. The PCR product was visualized by 1.2% agarose gel and stored at −20°C until further use.

### 2.5. Detection of Hepatitis Delta Virus (HDV) by ELISA

All the patient's sera positive for HBV DNA by PCR were checked for anti-HDV antibodies using 3rd generation ELISA assay kit (Globe Diagnostics, Italy) using the methodology described in the manufacturer's protocol. In brief, incubator was set to 37 ± 1°C. All the reagents were brought to room temperature before use (approximately 1 hour), without removing the plate from the bag. All components were shaken well. Then the plate was removed from the package. A 1/20 dilution of serum samples in tubes apart was prepared by adding 5 *μ*L of sample to 95 *μ*L of sample dilution solution (1/20 dilution). 80 *μ*L of sample dilution solution was added into all wells except in those assigned to controls. 20 *μ*L of the 1/20 dilutions of serum samples, 100 *μ*L of positive control, 100 *μ*L of cutoff (cutoff in duplicate), and 100 *μ*L of negative control were added into the corresponding wells. Plate was covered with a sealing sheet and incubated at 37 ± 1°C for 60 min. Then the antigen-conjugate complex was prepared. The seal was removed and aspirate liquid from all wells was washed five times with 0.3 mL of washing solution per well. Then Immediately 100 *μ*L of reconstituted antigen-conjugate complex was added into each well, covered with a sealing sheet, and incubated in incubator 37 ± 1°C for 60 min. The seal was removed and liquid from all wells was aspirated and washed five times with 0.3 mL of washing solution per well. Immediately after that 100 *μ*L of substrate solution was added into each well, incubated at room temperature for 20 min, and protected from light and then immediately 50 *μ*L of stopping solution was added into all wells, and finally read with ELISA Plate Reader at 450 nm within 1 hour of stopping.

### 2.6. Biochemical Factors

The following biochemical examinations were tested in present study. The level of fasting glucose, Total Cholesterol (TC), High-Density Lipoprotein (HDL), Alanine Amino Transferase (ALT), Aspartate Transaminase (AST), bilirubin, and fasting Triglyceride (TG) was measured by Microlab 300 apparatus (Merck). Sera of all 501 chronic HCV patients were analyzed for these parameters.

### 2.7. Statistical Analysis

In statistical analysis clinical variables presented as mean ± SD. Mean, SD, *P* value, and CI (95%) and standard error were calculated and compared by using independent *t*-test. For *P* value, the level of significance was 5%. Data analysis was accomplished using the computer software, SPSS Version 16.0 for windows.

### 2.8. Data Collection for Risk Factors

For the evaluation of common risk factors associated with the HCV transmission, no provincial data collection system exists. Data was collected by conducting interviews with each of the patients and using questionnaire.

## 3. Results

### 3.1. Molecular Screening of Samples

**Figure 1:**
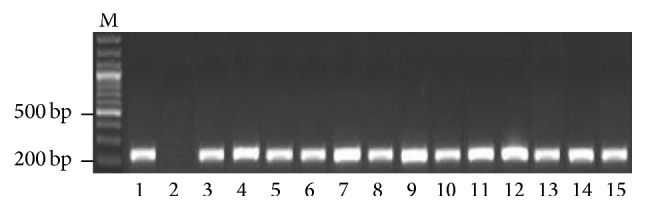
A representative 1.5% agarose gel of PCR products for the detection of HCV. Lane M: DNA marker, lane 1: positive control (227 bp), lane 2: negative control, and lanes 3 to 15: patients positive for HCV RNA.

A total of 730 samples, suspected for hepatitis C infection, were subjected to PCR-based detection for viral RNA, out of which 501 (with mean age of patients 39.35 ± 12.32) samples were proved as HCV positive, by giving a band size of approx. 227 bp in PCR, as shown in Figure 1.

### 3.2. Screening of HCV Positive Cases for HBV Infection (Coinfection)

**Figure 2:**
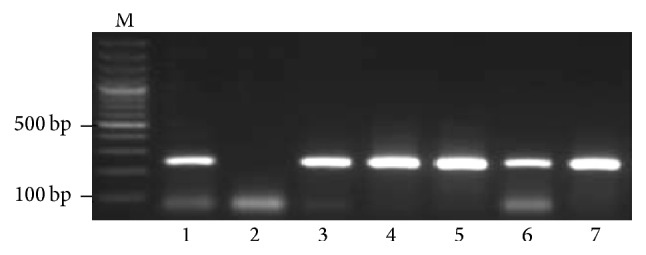
A representative 1.5% agarose gel of PCR products for the detection of HBV in HCV positive patients. Lane M: DNA marker, lane 1: positive control (242 bp), lane 2: negative control, and lanes 3 to 7: patients positive for HBV DNA.

A total of 501 HCV positive samples were screened for HBsAg through Immunochromatographic Technique (ICT HBsAg); out of 501 samples, 33 were (6.6%) found positive for HBsAg. These 33 samples were screened for HBV DNA through nested PCR technique and the results of nested PCR are shown in Figure 2. The positive samples gave a band size of approximate 242 bp. A total of 31 (6.2%) samples were found positive for HBV DNA and the data is given in Table 1.

### 3.3. Screening of HCV and HBV Coinfected Cases for HDV Infection

**Figure 3:**
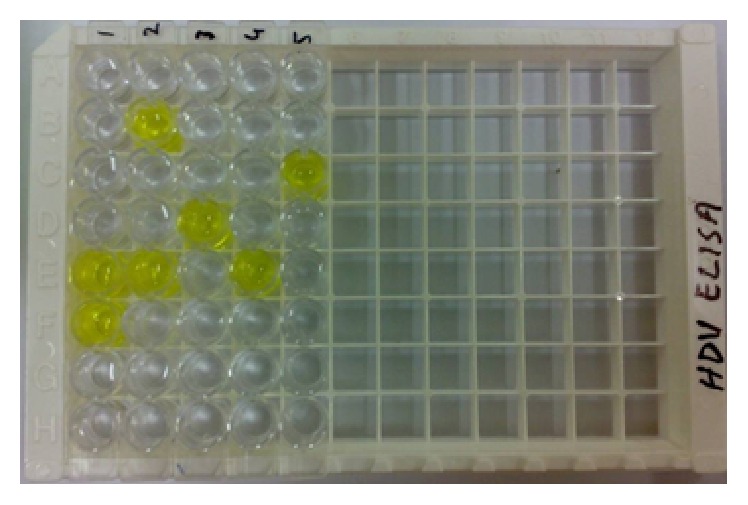
Anti-HDV Abs ELISA Plate: W 1A and 1B: blank, W 1C and 1D: negative controls, W 1E and 1F: positive controls, and W 2B, 2E, 3D, 4E, and 5C: anti-HDV Ab positive samples (W: Microtiter Well).

**Figure 4:**
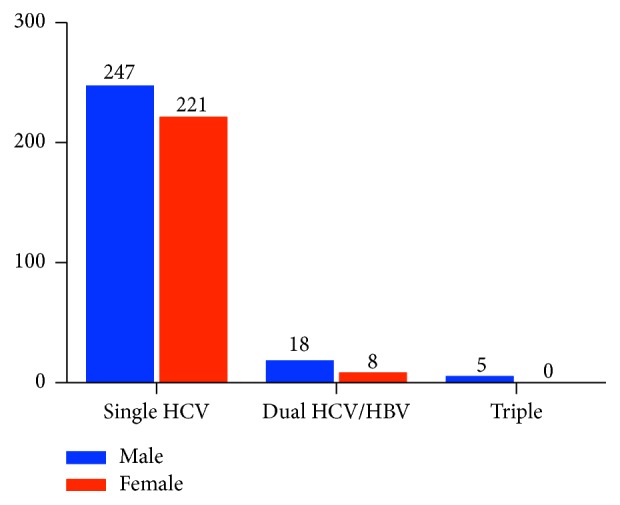
Bar graph representing the genderwise distribution of single, dual, and triple infections.

After the confirmation of samples for HBV DNA, 31 coinfected (HBV and HCV) samples were further screened for Hepatitis Delta (HDV) infection. Anti-HDV ELISA screening was performed in HBV and HCV coinfected samples and it was found that 5 (1% of the total HCV positive) samples were positive for anti-HDV antibodies showing that the 1% of the total 501 samples have triple infection of HCV, HBV, and HDV, with mean age of 30.20 ± 8.258, shown in Figures 3 and 4.

### 3.4. Biochemical Parameters (LFTS) of Single, Double, and Triple Infection Patients

The 501 samples were divided into 3 groups: (1) the patients with single infection (HCV), (2) patients with the double infection (HCV and HBV), and (3) patients with the triple infection (HCV, HBV, and HDV) were subjected to the analysis of liver function tests, that is, ALT, AST, and bilirubin. In case of single infection, the mean levels of AST, ALT, and bilirubin were found to be 76 IU/L, 91 IU/L, and 1.9 mg/dL, respectively. These levels were found to be 61 IU/L, 84 IU/L, and 1.6 mg/dL in case of double infection for AST, ALT, and bilirubin, respectively. The mean values of LFTs for triple infection were 174 IU/L, 348 IU/L, and 3.25 mg/dL, respectively (Table 2).

### 3.5. Evaluation of Potential Risk Factors Associated with the Transmission of Single, Double, and Triple Infections

Various possible risk factors, for example, blood transfusion, injectable drug users, dental operations, razor sharing, observed in the current study responsible for infection transmission with each single, double, and triple infection are given in Figures 6, 7, and 8 and Table 3.

### 3.6. Statistical Analysis of the Data

All the 501 patients were included in the study; 229 (46%) were females with mean age of 39.58 ± 11.07 and 229 (54%) were males with mean age of 39.15 ± 13.29. A summary of gender and age association of HCV infected patients is given in Table 4. It is evident from Table 4 that there is no significant relation between gender/mean age of the patients and HCV infection.

In the first group with HCV single infection alone, there was no significant difference between the number of males (53%) and number of females (47%) as well as the mean age of males (39.36 ± 13.37) and the mean age of females (39.80 ± 11.11) as shown in Table 5. Among the second group with dual hepatitis infection (HCV + HBV) the case was different from the first group. The numbers of males with dual infection were more than double (69%) as compared to females (31%). The mean age of females in this group was 33.5 ± 8.19 and that of males was 40.11 ± 13.02, as shown in Table 6. In the third group with triple hepatitis infection (HCV, HBV, and HDV) the observations were different. All of the patients in this group were males with mean age of 30.2 ± 8.25.

### 3.7. Comparison of LFTs of Patients with Coinfection (HCV/HBV) and Single Infection (HCV)

**Figure 5:**
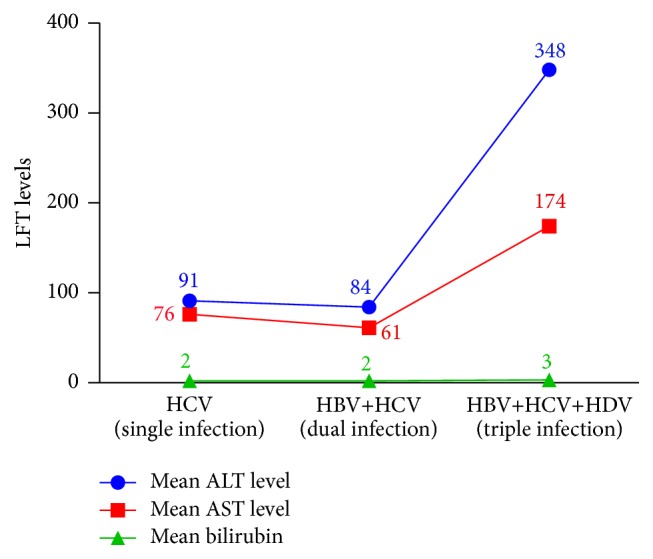
Comparison of liver function test results among HBV, HCV, and HDV positive cases.

LFTs of coinfected patients (HCV and HBV) were compared with HCV infected patients through independent *t*-test. It was found that the LFTs of dually infected patients were significantly lower (closer to the normal values) as compared to the single infected patients with *P* values 0.020, <0.05, and 0.052 for ALT, AST, and bilirubin, respectively, as shown in Figure 5.

Then the LFTs of patients with triple infection (HCV, HBV, and HDV) were compared with single infected patients (HCV) through independent *t*-test. It was found that the LFTs of triple infected patients were significantly higher (away from the normal values) as compared to the single infected patients with all the three *P* values <0.05 for ALT, AST, and bilirubin, as shown in Figure 5.

The study subjects were divided into three groups. In the first group, patients having single infection with HCV were placed, and the second group comprised patients having double infection with HCV and HBV. The patients who were infected with all three viruses were placed in the third group.

### 3.8. Possible Associated Routes of Transmission in Coinfection

**Figure 6:**
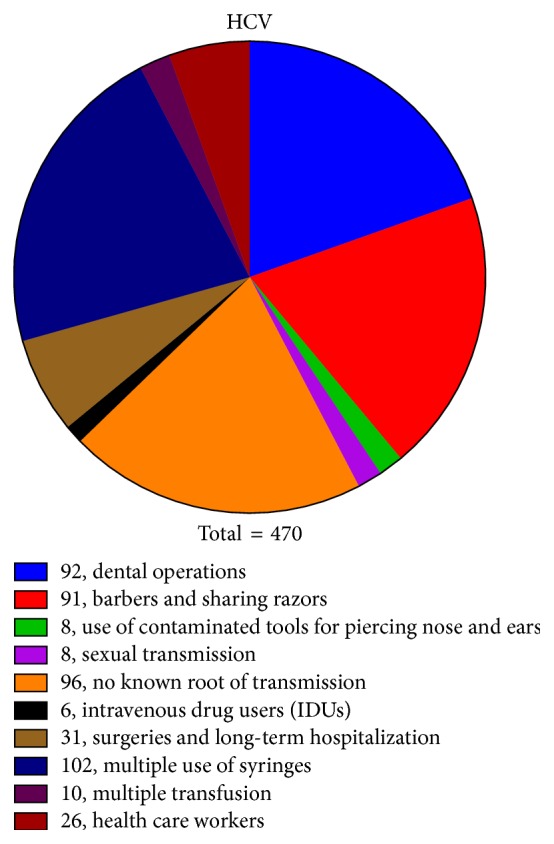
Risk factors associated with infection transmission in HCV single infection.

**Figure 7:**
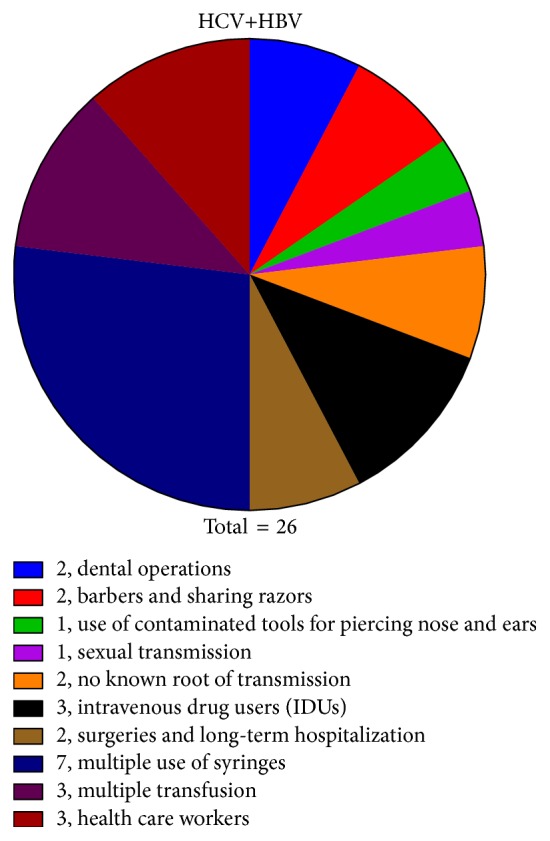
Risk factors associated with HCV and HBV coinfection.

**Figure 8:**
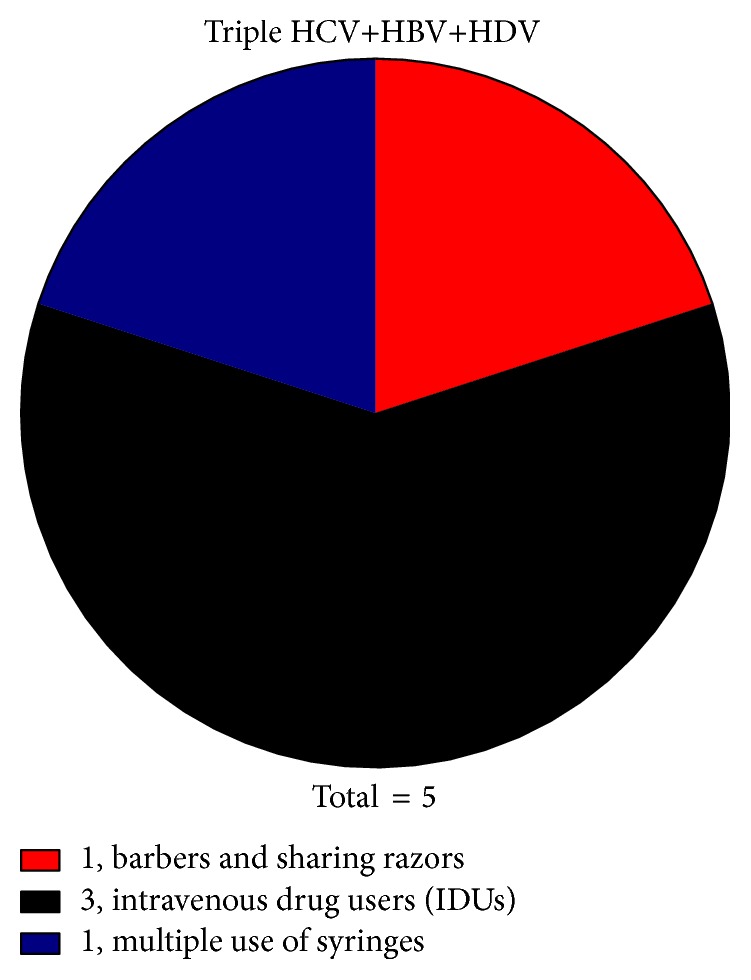
Risk factors associated with HCV, HBV, and HDV triple infection.

In order to establish an association of coinfection (either double or triple) with route of transmission, the whole data of routes of transmission were divided into 10 groups comparing routes between coinfected and single infected patients. After analyzing the data, it was found that the most important route of transmission associated with coinfection was intravenous drug users (IDU) in which 50% (6/12) of the subjects were found coinfected with HBV and HDV. From this data, the other possibly associated route of transmission for the coinfection may be the multiple blood transfusions with 23% (3/13) of the coinfected cases. In the case of sexual transmission, use of contaminated tools for nose and ear piercing (11.1% of coinfected patients in each) and health care workers (10.3%) may also be considered as the associated routes of transmission for the coinfection as shown in Figures 6, 7, and 8.

## 4. Discussion

The coinfection of HBV in HCV patients is frequent in Pakistan and this study indicates that superinfection with HDV is also a serious problem. Chronic hepatitis due to different hepatic viruses is a common cause of liver related morbidity. Hepatitis B (HBV) and hepatitis C (HCV) are the main causes for chronic hepatitis [1, 24, 25]. It has been shown that superinfection of hepatitis A or E over HBV or HCV could lead to patient's deterioration and increase or precipitate encephalopathy [26]. Infection with multiple viruses leads to management problems with higher incidence of morbidity and mortality [5]. Presence of dual and triple viral infections has been reported from various parts of the world. As hepatitis B, hepatitis C, and hepatitis D share same modes of transmission, infection with more than one virus is possible [6]. The objectives of the current study were to determine the double and/or triple viral infections of hepatitis B and hepatitis D in chronic HCV patients, the effect(s) of triple infection (if present) of hepatitis on liver, and risk factors involved in its transmission.

In the present study, 501 HCV positive samples, 54.3% males and 45.7% females, were screened for dual infection of HBV and 31 (6.2%) were found positive for dual infection. The dually infected patients were screened for the presence of HDV and 5 (1%) were found positive for triple infection. In present study, the HBV DNA was detected in 55.2% of patients followed by HCV in 24.1%. This effect could be due to the fact that the authors primarily inducted the patients for hepatitis B diagnosis and tested them for HDV and HCV while they primarily inducted the patients of chronic hepatitis and tested for all three viruses. Infections were predominantly acquired through injection of drugs (i.e., HBV and HCV). In case of liver infection, the levels of ALT, AST, and bilirubin usually rise up indicating liver injury. The mean value of AST, ALT, and bilirubin levels in the patients with chronic hepatitis C alone was found to be 76 ± 24.88 IU/L, 91 ± 45.38 IU/L, and 1.9 ± 0.69 mg/dL, respectively. In case of coinfection of HBV in HCV patients, the mean AST, ALT, and bilirubin level were found to be 61 (±15.39), 84 (±16.26), and 1.6 (±0.73), respectively. The level of AST and ALT enzymes and bilirubin in triple hepatitis was comparatively higher as compared to single/dual infection. LFTs (Figure 5 and Table 2) were 174 (±77.00), 348 (±117.05), and 3.25 (±0.88). It was found that multiple use of syringes (110) (22%) and dental operation (94) (18.7%) are the major route of transmission in case of HCV alone or coinfection of HBV, while the use of contaminated needles is the standalone cause of triple hepatitis affecting 80% of patients in the study subjects. Large proportion of patients enrolled in the study (96) (20.4%) even did not know how they acquire these viral infections. This might be due to lack of awareness of viral hepatitis, poor sanitary condition, and lack of proper disposal of waste material. Nearly in all samples, HCV was acquired via IDU; HCV seroprevalence was 89.7% among injection drug users, compared with 10.5% among noninjection drug users, and IDU was by far the strongest risk factor for HCV infection in multivariate analyses.

## Figures and Tables

**Table 1:** Coinfection rate of HBV and HCV.

	Number	Mean age	Std. deviation	*P* value
Non-coinfected	469	39.42	12.346	0.589
Coinfected with HBV	31	38.08	11.996	

**Table 2:** Comparison of liver function test results among HBV, HCV, and HDV positive cases.

	Liver function tests			
	Mean AST	Mean ALT	Mean bilirubin	(*N*) Positive
Ref. range	9–40 IU/L	10–40 IU/L	0.2–1.0 mg/dL	
HCV (single infection)	76 (±24.88)	91 (±45.83)	1.9 (±0.69)	470
HBV and HCV (dual infection)	61 (±15.39)	84 (±16.26)	1.6 (±0.73)	26
HBV, HCV, and HDV (triple infection)	174 (±77.00)	348 (±117.05)	3.25 (±0.88)	5

**Table 3:** Possible associated risk factors for infection transmission.

Risk factors	Cases			
	HCV (total = 470)	HCV and HBV (total = 26)	HCV, HBV, and HDV (total = 5)	Total (501)
Barbers and sharing razors	91 (19.3%)	2 (7.6%)	1 (20%)	94 (18.7%)
Dental operations	92 (19.5%)	2 (7.6%)	0	94 (18.7%)
Health care workers	26 (5.5%)	3 (11.5%)	0	29 (5.7%)
Multiple transfusion	10 (2.12%)	3 (11.5%)	0	13 (2.6%)
Multiple use of syringes	102 (21.7%)	7 (27.0%)	1 (20%)	110 (22.0%)
Surgeries and long-term hospitalization	31 (6.6%)	2 (7.6%)	0	33 (6.6%)
Intravenous drug users (IDUs)	6 (1.2%)	3 (11.5%)	3 (60%)	12 (2.4%)
No known root of transmission	96 (20.4%)	2 (7.6%)	0	98 (19.5%)
Sexual transmission	8 (1.7%)	1 (3.9%)	0	9 (1.8%)
Use of contaminated tools for piercing nose & ears	8 (1.7%)	1 (3.9%)	0	9 (1.8%)

**Table 4:** Total patients in study (age and gender).

Gender	*N*	Mean age	Std. deviation	*P* value
Female	229 (46%)	39.58	11.078	0.070
Male	272 (54%)	39.15	13.294	

**Table 5:** Patients with single infection (age and gender).

Gender	*N*	Mean age	Std. deviation	*P* value
Female	221 (47%)	39.8009	11.11985	0.703
Male	247 (53%)	39.3644	13.37532	

**Table 6:** Patients with dual infection (age and gender).

Gender	*N*	Mean age	Std. deviation	*P* value
Female	8 (31%)	33.5000	8.19407	0.201
Male	18 (69%)	40.1111	13.02888	
